# Antimicrobial, Antidiabetic, Antioxidant, and Anticoagulant Activities of *Cupressus sempervirens* In Vitro and In Silico

**DOI:** 10.3390/molecules28217402

**Published:** 2023-11-02

**Authors:** Aisha M. H. Al-Rajhi, Marwah M. Bakri, Husam Qanash, Hassan Y. Alzahrani, Haneen Halawani, Meaad A. Algaydi, Tarek M. Abdelghany

**Affiliations:** 1 Department of Biology, College of Science, Princess Nourah bint Abdulrahman University, Riyadh 11671, Saudi Arabia; amoalrajhi@pnu.edu.sa; 2 Biology Department, College of Science, Jazan University, Jazan 82817, Saudi Arabia; mabakri@jazanu.edu.sa; 3 Department of Medical Laboratory Science, College of Applied Medical Sciences, University of Ha’il, Hail 55476, Saudi Arabia; 4 Medical and Diagnostic Research Center, University of Ha’il, Hail 55473, Saudi Arabia; 5 University Medical Service Center, King Abdulaziz University, Building 70, Jeddah 21589, Saudi Arabia; hyalzahrani1@kau.edu.sa (H.Y.A.); hkhalwani@kau.edu.sa (H.H.); malqaedi@kau.edu.sa (M.A.A.); 6 Botany and Microbiology Department, Faculty of Science, Al-Azhar University, Cairo 11725, Egypt

**Keywords:** *Cupressus sempervirens*, microbial inhibition, antioxidant, antidiabetic, anticoagulant

## Abstract

In the last decade, the urgent need to explore medicinal plants or drug development has increased enormously around the world to overcome numerous health problems. In the present investigation, HPLC indicated the existence of 18 phenolic and flavonoid compounds in the *Cupressus sempervirens* extract. Hesperetin represents the greatest concentration (25,579.57 µg/mL), while other compounds, such as pyro catechol, rutin, gallic acid, chlorogenic acid, naringenin, and quercetin, were recognized in concentrations of 2922.53 µg/mL, 1313.26 µg/mL, 1107.26 µg/mL, 389.09 µg/mL, 156.53 µg/mL, and 97.56 µg/mL, respectively. The well diffusion method documented the antibacterial/antifungal activity of *C. sempervirens* extract against *E. faecalis*, *E. coli*, *C. albicans*, *S. typhi*, *S.aureus*, and *M. circinelloid* with 35, 33, 32, 25, 23, and 21 mm inhibition zones, respectively, more than the standard antibiotic/antifungal agent. Low values ranging from 7.80 to 15.62 µg/mL of MIC and MBC were recorded for *E. faecalis*, *E. coli*, and *C. albicans*. From the 1- diphenyl-2-picryl hydrazyl (DPPH) assay, promising antioxidant activity was recorded for *C. sempervirens* extract with IC_50_ of an 8.97 µg/mL. Moreover, ferric reducing antioxidant power (FRAP) and total antioxidant capacity assays (TAC) confirmed the antioxidant activity of the extract, which was expressed as the ascorbic acid equivalent (AAE) of 366.9 ± 0.2 µg/mg and 102 ± 0.2 µg/mg of extracts, respectively. α-amylase and α-glucosidase inhibition % were determined to express the antidiabetic activity of the extract in vitro, with promising IC_50_ value (27.01 µg/mL) for α-amylase compared to that of acarbose (50.93 µg/mL), while IC_50_ value of the extract for α-glucosidase was 19.21µg/mL compared to that of acarbose 4.13 µg/mL. Prothrombin time (PT) and activated partial thromboplastin time (APTT) revealed the role of *C. sempervirens* extract as an anticoagulant agent if compared with the activity of heparin. Binding interactions of hesperetin and gallic acid were examined via the Molecular Operating Environment (MOE) Dock software against *E. faecalis* (PDB ID: 3CLQ), *C. albicans* (PDB ID: 7RJC), α-amylase (PDB ID: 4W93), and α-glucosidase (PDB ID: 3TOP). The obtained results shed light on how molecular modeling methods might inhibit the tested compounds, which have the potential to be useful in the treatment of target proteins.

## 1. Introduction

Today, drug development or discovery is emphasized by scientific researchers to overcome multidrug-resistance microorganisms and dispersed diseases, which are considered global problems. A wide variety of phytoconstituents play numerous biological functions [1,2,3,4,5]. *Cupressus sempervirens* is an evergreen tree, one of the traditional aromatic and medicinal plants that contain a wide range of bioactive metabolites. It belongs to the Cupressaceae family, and it is a species of cypress native to the eastern Mediterranean region. Phytochemical screening of *C. sempervirens* indicated the existence of several classes of chemical compounds, such as phenols, flavonoids, alkaloids, and terpenoids [6]. As mentioned by Selim et al. [7], analysis of constituents indicated the presence of 0.7% alkaloids, 0.22% flavonoids, 0.31% tannin, 1.9% saponins, and 0.067% phenols besides some essential oils with several other biologically active compounds from *C. sempervirens*. Several pharmacological applications were associated with *C. sempervirens* extract, such as memory improvement, neuroprotection [8], stimulation of venous circulation to the area of the bladder and kidney, and elimination of fluid retention [9], as well as having antibacterial, antiparasitic, antifungal, antioxidant, antiviral, insecticidal, wound healing, anticoagulant, anticancer, and estrogenic properties [10]. Moreover, it was used for the treatment of neurodegenerative disorders and diabetes mellitus type 2 [11]. Additionally, the antileishmanial potential of *C. sempervirens* extract was reported against different forms, including amastigote and promastigote of *Leishmania major* and *L. infantum* [12]. Anticancer activity either in vitro or in vivo with large antiproliferative activity [13], astringent, antiinflammatory, aromatherapeutic, antispasmodic, antiperspirant, and diuretic properties [14] of *C. sempervirens* extract was documented.

*C. sempervirens* reflected antimicrobial activities, but the solvent used represents one of the most important factors that affects the level of the inhibitory potential of the *C. sempervirens* extract. For instance, Selim et al. [7] reported strong antibacterial activity but not antiyeast activity using methanolic *C. sempervirens* extract, while ethanolic extract showed moderate antibacterial activity. Moreover, *Klebsiella pneumoniae* was the most sensitive to methanolic extract with complete killing of bacterial cells when exposed to 250/500 μg/mL at 30/120 min using ethanolic/ethanolic extract. Azzaz et al. [15] mentioned the inhibitory potential of cypress oil against two fungi, *Verticillium* and *Aspergillus*. Different microorganisms were exposed to the essential oil of *C. sempervirens*, some of which, including *Escherichia coli*, *Staphylococcus aureus*, *Bacillus subtilis*, *Aspergillus niger*, and *Candida albicans*, were inhibited, but with different inhibition levels, where *Bacillus subtilis* was found to be the most susceptible, while *E. coli* was resistant, and a moderate effect was observed on *Staphylococcus aureus*, *Candida albicans*, and *Aspergillus niger* [16]. Previously, Asgary et al. [17] focused on the antioxidant power of chloroformic and methanolic extracts of *C. sempervirens*. Furthermore, Shahid et al. [18] have documented the antioxidant potential of *C. sempervirens* leaves extracted by methanol. Recently, Galovičová et al. [19] reported several biological activities of the essential oil extracted from *C. sempervirens*, such as antibacterial (against *Pseudomonas aeruginosa*, *Salmonella enterica*, *Yersinia enterocolitica*, *Bacillus subtilis*, *Enterococcus faecalis*, and *Staphylococcus aureus*) antifungal (against *Candida krusei*, *C. albicans*, *C. tropicalis*, *C. glabrata*, *Penicillium citrinum*, *Aspergillus flavus*, and *Botrytis cinerea*), antioxidant, and anticancer. Computational approaches are currently an imperative tool for providing information about the action mechanisms of biologically active compounds. From this approach, molecular docking, which permits the user to dock the active constituents with the active site of the target in the living system [3]. Molecular Operating Environment (MOE), a powerful molecular visualization software and considered a molecular modeling program, is particularly designed to deal with numerous biological compounds. Moreover, it is applied to predict the binding sites and docking score. The root-mean-square deviation (RMSD) is used to detect the differences among true or predicted values of biological compounds. Furthermore, the fields of RMSD and RMSD-refine were applied to compare the results pose-with-pose in the co-crystal ligand position before and after amendment, respectively [1,3]. In the current decade, molecular docking studies have attracted the attention of biologists to assess the affinity of any natural or chemical molecules in relation to a particular biological target. Moreover, it permits decreasing the time and minimizing the cost that required to perform the same biological activity mechanisms [15,16]. The natural constituents originating from plants can be repurposed by these approaches to prove their applications [20]. This investigation aimed to assess the antimicrobial, antidiabetic in vitro and in silico, antioxidant, and anticoagulant potential of *C. sempervirens* extract.

## 2. Results and Discussion

### 2.1. Phytochemical Characterization of C. sempervirens Extract

**Figure 1:**
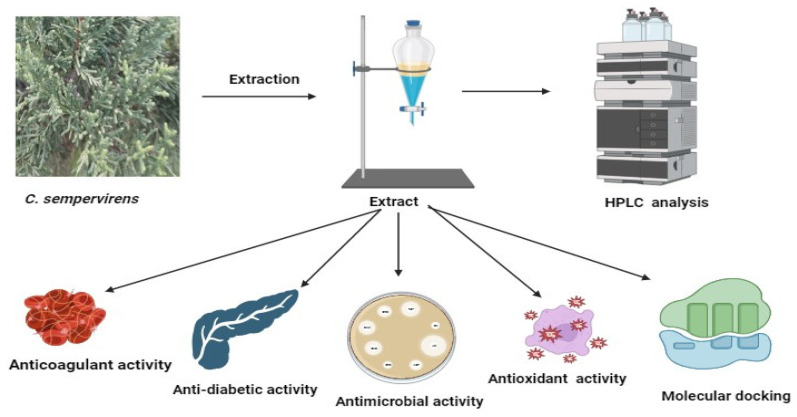
Diagrammatic form illustrating experienced processing on *C. sempervirens*. This shape was generated via BioRender.com. (accessed on 30 May 2023).

**Figure 2:**
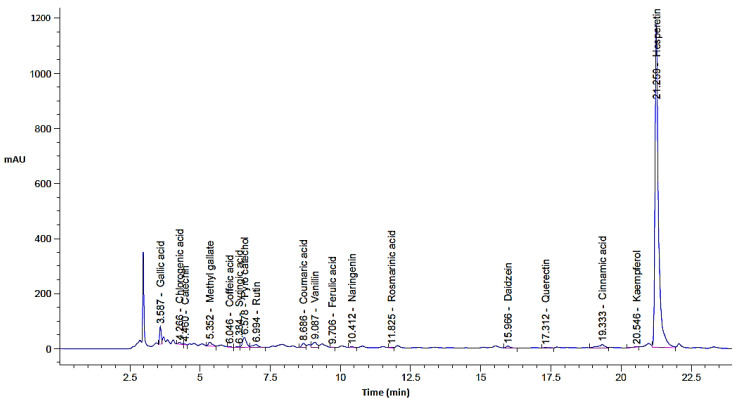
*C. sempervirens* extract chromatogram analysis by HPLC for phenolic and flavonoid detection.

In the current investigation, the *C. sempervirens* plant was extracted and subjected to phenolic and flavonoid analysis via HPLC, with multiple biological utilizations as summarized in Figure 1. From HPLC analysis, 18 compounds associated with phenolics and flavonoids were detected in the *C. sempervirens* extract. The detected compounds differed in retention time, area, area %, and concentration (Table 1 and Figure 2). A promising concentration (25,579.57 µg/mL) of hesperetin was recognized in the *C. sempervirens* extract, followed by pyro catechol (2922.53 µg/mL), rutin (1313.26 µg/mL), and gallic acid (1107.26 µg/mL). Other important constituents were identified, such as chlorogenic acid (389.09 µg/mL), naringenin (156.53 µg/mL), rosmarinic acid (145.71 µg/mL), and quercetin (97.56 µg/mL). The concentration of ferulic acid in the *C. sempervirens* extract was low (11.53 µg/mL); at the same time, ellagic acid was injected as a standard in HPLC but not detected in the *C. sempervirens* extract. Previous results revealed the existence of ten flavonoids with high quantities in *C. sempervirens* leaves extracted by ethyl acetate such as narengin, hesperetin, quercetrin, and rutin, besides 24 phenolic compounds with a high quantity of vaniliic, coumaric, salicylic, ferulic, and pyrogallol [21], while the present investigation showed less number of detected compounds, may be due to the extraction solvent, climatic, and nutrient conditions of the cultivated plant. The identified compounds in the *C. sempervirens* extract possess numerous biological activities, as mentioned in previous reports [22,23,24,25,26,27]. For instance, naringin exhibited antiinflammatory and antioxidant activities [22]. Proliferation of several cancer cells such as breast, prostate colon, and lung were prevented using rutin [23]. Srivastava et al. [24] demonstrated the anticancer activity of quercetin. Multi-functions were reported to chlorogenic acid, such as liquefy blood clot [25], hypoglycemic antioxidant, and antiinflammation [26]. In the present study, kaempferol was detected in the *C. sempervirens* extract, and as mentioned previously [27], it acts as an antioxidant by decreasing oxidative stress as well as improving the efficacy of insulin via decreasing insulin resistance and preserving the shape and activity of pancreatic β-cell.

### 2.2. Antimicrobial Activity of C. sempervirens Extract

**Figure 3:**
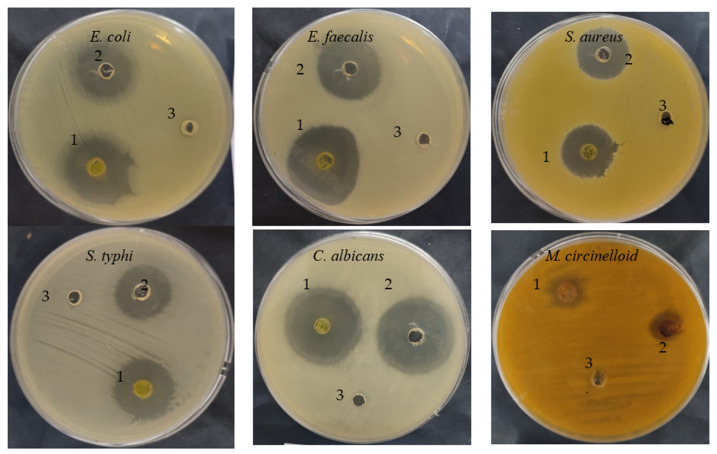
Antimicrobial activities of *C. sempervirens* extract (1), antibiotic/antifungal (2), negative control (3).

The *C. sempervirens* extract demonstrated antibacterial/antifungal activity against various bacteria/fungi (Table 2 and Figure 3), but with different levels of inhibition zone (IZ) depending on the microbial species. For instance, *E. faecalis* was the most sensitive (35 mm IZ), followed by *E. coli* (33 ± 0.2 mm IZ), *C. albicans* (32 ± 0.2 mm IZ), *S. typhi* (25 ± 0.1 mm IZ), *S. aureus* (23 ± 0.4 mm IZ), and *M. circinelloid* (21 ± 0.2 mm IZ). These differences may be due to cell wall composition, the presence of capsules, the ability of microorganisms to produce biofilm, and genes responsible for resistance to antimicrobial agents. Additionally, the microbial inhibitory potential of our collected plant may differ from other studies, where the phytoconstituents and biological activities may differ in the same species of plant depending on cultivation area, climatic conditions, and nutritional conditions. Sriti et al. [28] investigated the essential oil contents, antioxidant, and antimicrobial activities of *C. sempervirens* L. collected from three regions, including Bizerte, Ben-Arous, and Nabeul. The plant collected from Bizerte and Ben Arous possesses a higher content of essential oils (0.56%), followed by the plant collected from Nabeul (0.49%). High antioxidant activity was attributed to the essential oil of collected plant from Bizerte with an IC_50_ value of 55 µg/mL, followed by essential oil of the plant collected from Ben-Arous with an IC_50_ value of 97.50 µg/mL and Nabeul with an IC_50_ value of 155 µg/mL. The essential oil of the plant collected from Bizerte was the most effective against *E. faecalis*, with a 65 mm of inhibition zone. In the current research, the inhibitory potential of *C. sempervirens* extract was greater than the inhibitory potential of standard antibiotic and antifungal agents against all tested microorganisms. The MIC and MBC values were lower for *E. faecalis*, *E. coli*, and *C. albicans*, ranging from 7.80 ± 0.5 to 15.62 ± 1.33 µg/mL, while the MIC and MBC against *S. aureus* were 125 µg/mL. On the other hand, the values of MIC and MBC against *M. circinelloid* were high, i.e., 125 ± 3.0 and 500 ± 5.0 µg/mL, respectively. Therefore, from the values of MIC/MBC index, it is clear that *C. sempervirens* extract reflected cidal effects against tested organisms, except *M. circinelloid*, because all values were less than 4. The ethyl acetate extract of *C. sempervirens* flowers exhibited antibacterial activity against *Bacillus cereus* with an MIC of 100 μg/mL and *S. aureus* with an MIC of 50 μg/mL [12]. Recently, Yazdani et al. [29] studied the effect of *C. sempervirens* extracted by methanol on various bacteria; they reported that MIC and MBC were 125 μg/mL and 250 μg/mL against *S. aureus*; 250 mg/mL and 500 μg/mL against *B. cereus*, respectively. The differences among our results and the findings of other investigators may be explained according to the different origins of *C. sempervirens* extract and its acquisition technique.

### 2.3. Antioxidant Activity of C. sempervirens Extract

**Figure 4:**
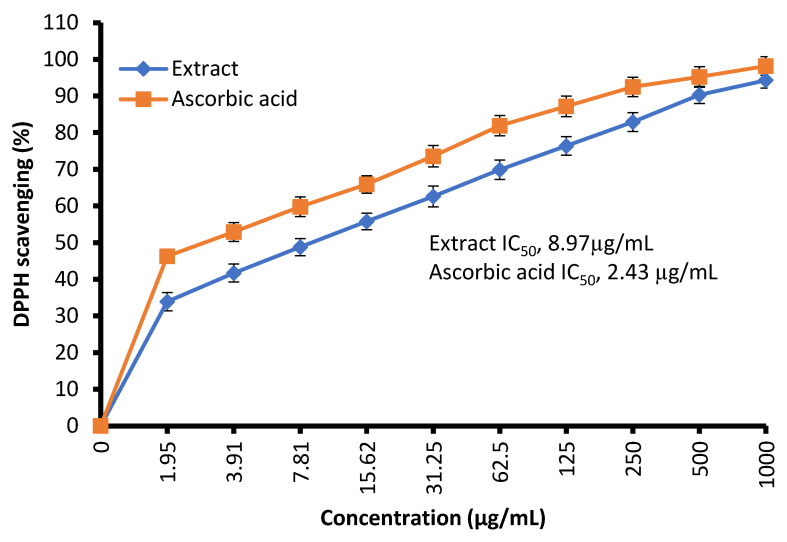
Antioxidant activity of *C. sempervirens* extract using DPPH assay.

**Figure 5:**
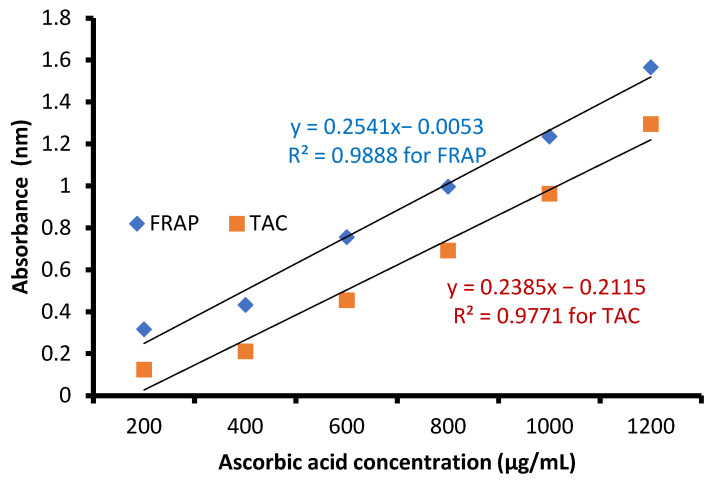
Standard curve of ascorbic acid.

The free radical-scavenging potential of the tested extract and ascorbic acid as a positive control are illustrated in Figure 4. In fact, *C. sempervirens* extract increased the inhibition of scavenging % with effectiveness IC_50_ of 8.97 µg/mL compared to the IC_50_ value of ascorbic acid (2.43 µg/mL). Previously, *C. sempervirens* essential oils exhibited antioxidant activity with an IC_50_ of 151 µg/mL using DPPH [30]. The existence of constituents with diverse functional groups in extracts, besides the analytical technique utilized can lead to differing results. The appeared antioxidant potential of *C. sempervirens* extract by different protocols indicated that the natural constituents of the extract work synergistically to create a wide range of antioxidant properties. The methanolic extract of *C. sempervirens* cultivated in Egypt possessed strong DPPH radical-scavenging potential, as described previously [16]. On the contrary, the methanolic extract of *C. sempervirens* cultivated in Turkey had a moderate scavenging activity [8], which might be depending on their flavonoid and phenolic content. Different extraction solvents of *C. sempervirens* including methanol, chloroform, ethylacetate, and hexane exhibited antioxidant activities, but with different IC_50_ values of 39.25, 105.63, 64.07, and 85.62 µg/mL, respectively [31]. The antioxidant activity of the extract was documented also by FRAP, which expressed as ascorbic acid equivalent (AAE) 366.9 ± 0.2 µg/mg (data not tabulated) through standard curve of ascorbic acid (Figure 5). As mentioned previously, the reducing properties are linked with the occurrence of constituents, which appear to have their action via hydrolyze the free radical chain across the offering atom of hydrogen [32]. This suggested the existence of inhibiting constituents because of FRAP activity in the current extract of *C. sempervirens*. Another method via TAC confirmed the antioxidant activity of *C. sempervirens* extract, which was expressed as ascorbic acid equivalent (AAE) 102 µg/mg of extract (data not tabulated) through the standard curve of ascorbic acid (Figure 5).

In the current investigation, the antioxidant activity of *C. sempervirens* extract may be due to the occurrence of numerous phenolic and flavonoid compounds, as shown via HPLC analysis. Therefore, according to published investigations, a greatly positive association among the phenolic compounds and antioxidant potential seems to be the trend in several species of plants [33,34,35,36]. Flavonoids may capture peroxide, superoxide, and hydroxyl radicals, which may be one of the mechanisms of oxidative damage prevention or repress the proteins responsible for the formation of free radicals. Antioxidant activity of *C. sempervirens* extract using eight solvents was recorded [21], where DPPH scavenging potential ranged from 8.75% to 95.43%. Therefore, in the same study, *C. sempervirens* extract was applied to minimize hepatotoxicity and oxidative damage in rat’s liver. Zengin et al. [11] showed that *C. sempervirens* exhibited excellent antioxidant power compared to other studied plants, including *Artemisia absinthium* and *Lippia triphylla*.

### 2.4. Antidiabetic Activity of C. sempervirens Extract

**Figure 6:**
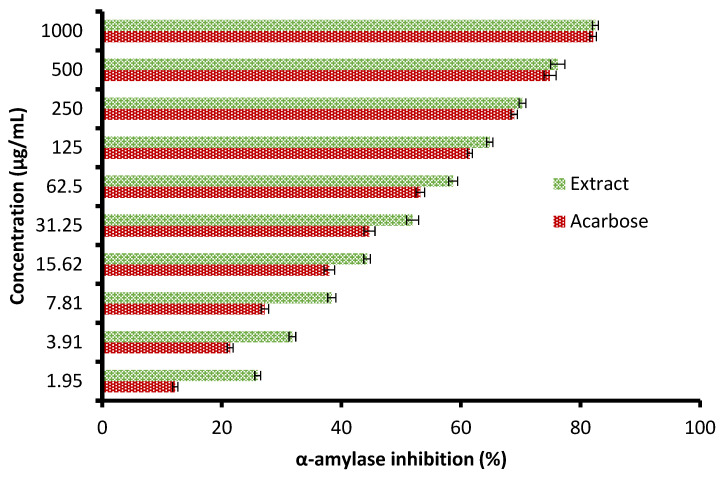
Antidiabetic properties of *C. sempervirens* extract via measuring α-amylase.

**Figure 7:**
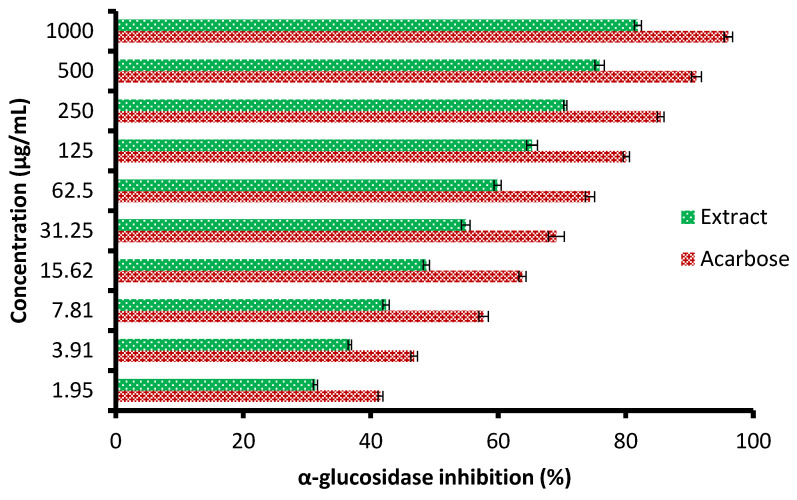
Antidiabetic properties of *C. sempervirens* extract via measuring of α-glucosidase.

Currently, the management of diseases such as diabetes by pharmaceutical plants is recommended due to their high contents of various saponins, flavonoids, terpenoids, alkaloids, carotenoids, and glycosides, which may enjoy antidiabetic properties. The height of glucose levels in the blood and hyperglycemia are greatly affected by the extent of some oxidative enzymes, including α-glucosidase and α-amylase. These enzymes encourage the metabolic reactions responsible for the analysis of complex saccharides into simple sugars such as glucose and its metabolized rate [22]. The antidiabetic potential of the *C. sempervirens* extract was evaluated by measuring α-amylase (Figure 6) and α-glucosidase (Figure 7) inhibition percentages. The inhibition of both α-amylase and α-glucosidase was increased with increasing the tested quantity of the extract. At concentrations from 62.5 up to 1000 µg/mL of the extract, negligible differences between α-amylase and α-glucosidase inhibition appeared, where the inhibition was 58.7 ± 1.33 and 59.9 ± 1.2% at 62.5 µg/mL; 70.3 ± 2.66 and 70.5 ± 0.75% at 250 µg/mL; and 82.5 ± 2.25 and 81.9 ± 1.00% at 1000 µg/mL, respectively. While at low concentrations of 1.95 up to 31.25 µg/mL, remarkable differences were visualized between α-amylase and α-glucosidase inhibition, where the inhibition was 26.0 ± 0.33 and 31.3 ± 0.50% at 1.95 µg/mL; 31.8 ± 1.33 and 36.7 ± 0.78% at 3.91 µg/mL; and 51.9 ± 1.5 and 54.9 ± 1.65% at 31.25 µg/mL, respectively. Surprisingly, the IC_50_ value of the extract (27.01 ± 1.0 µg/mL) was lower than that of acarbose (50.93 ± 0.5 µg/mL) for α-amylase, while the IC_50_ value of the extract (19.21 ± 0.33 µg/mL) was more than that of acarbose (4.13 ± 0.25 µg/mL) for α-glucosidase. These results indicated the presence of different mechanisms of enzyme inhibition by the extract and acarbose. However, generally, from the obtained findings, the IC_50_ values of the extract indicated its antidiabetic properties. Moreover, the differing IC_50_ value of *C. sempervirens* extract for enzymes inhibition in our study may be due to explanation of Truscheit et al. [33], as mentioned previously, who stated that α-amylase generally joined longer than α-glucosidase with polysaccharides, and acarbose is a pseudotetrasaccharide comprising a non-hydrolyzable nitrogen-jointed bond that represses the activity of α-amylase via competitive as reversible represses. According to the results of Shaki et al. [34], the reduction of glucose in the blood and the management of diabetes have occurred using *C. sempervirens* extract. *C. sempervirens* among two plants, including *Artemisia absinthium* and *Lippia triphylla* [11], caused the highest inhibition of α-amylase and α-glucosidase.

### 2.5. Anticoagulant Activity of C. sempervirens Extract

**Figure 8:**
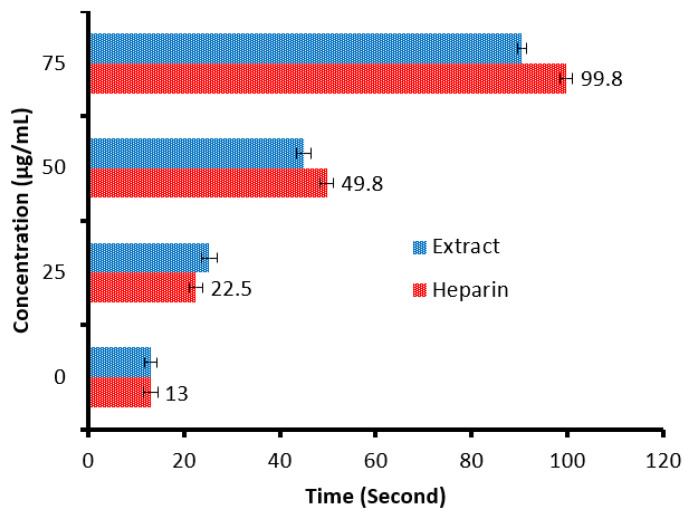
Anticoagulant activity of *C. sempervirens* extract and heparin at different concentrations via prothrombin time (PT).

**Figure 9:**
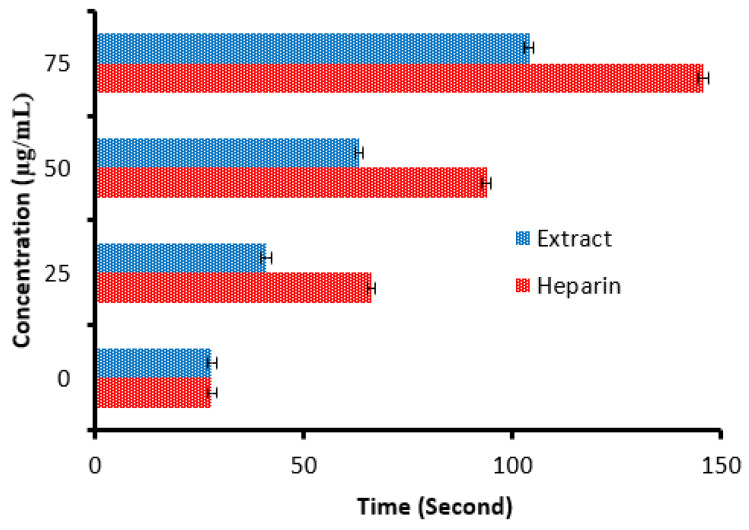
Anticoagulant activity of *C. sempervirens* extract and heparin at different concentrations via activated partial thromboplastin time (APTT).

There is a remarkable increment in the clotting time of PT and APPT values of plasma treated with the *C. sempervirens* extract at all tested concentrations (Figure 8 and Figure 9), where at 25, 50, and 75 µg/mL, the clotting time was 25.3 ± 0.29, 44.9 ± 1.0, and 90.5 ± 2.55 s of PT (Figure 8), and 41 ± 2.05, 63.2 ± 1.33, and 104.1 ± 3.25 s of APPT (Figure 9), respectively. The prolongation of clotting time may support the idea that the influence of *C. sempervirens* extract activates optimally within a limited dose range. Therefore, prolonged PT and PTT by *C. sempervirens* extract management propose inhibition of factors V, X, and prothrombin of the common pathway of coagulation. These results, if compared with heparin, are considered very promising in case of the PT test and in the case of the APPT test, where the clotting time was 22.5 ± 0.33, 49.8 ± 1.21, and 99.8 ± 2.66 s for PT, and 66.1 ± 2.3, 93.8 ± 2.15, and 145.7 ±2.33 s for APPT at 25, 50, and 75 µg/mL, respectively (the PTT reference range is 30–40 s, while the reference range of the PTT is 60–70 s). The obtained findings revealed the anticoagulant properties of *C. sempervirens* extract and may encourage the dissolution of clots. The anticoagulant potential of *C. sempervirens* extract could be due to the occurrence of some natural compounds as recorded by HPLC analysis in the present investigation. The anticoagulant properties of *C. sempervirens* were reported to be anticoagulant [14].

### 2.6. Molecular Docking Interaction of Hesperetin and Gallic Acid with Microbial Proteins, α-Amylase and α-Glucosidase

**Figure 10:**
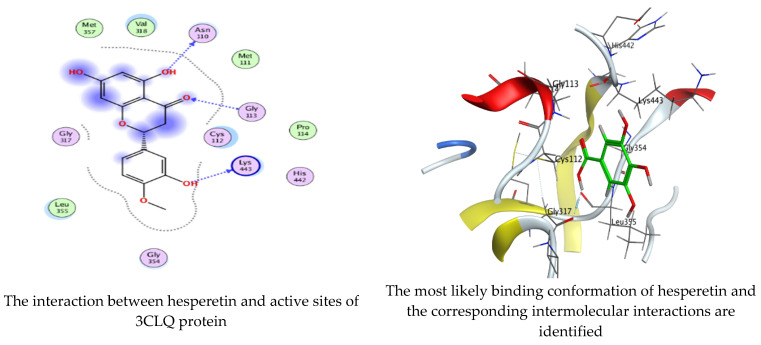

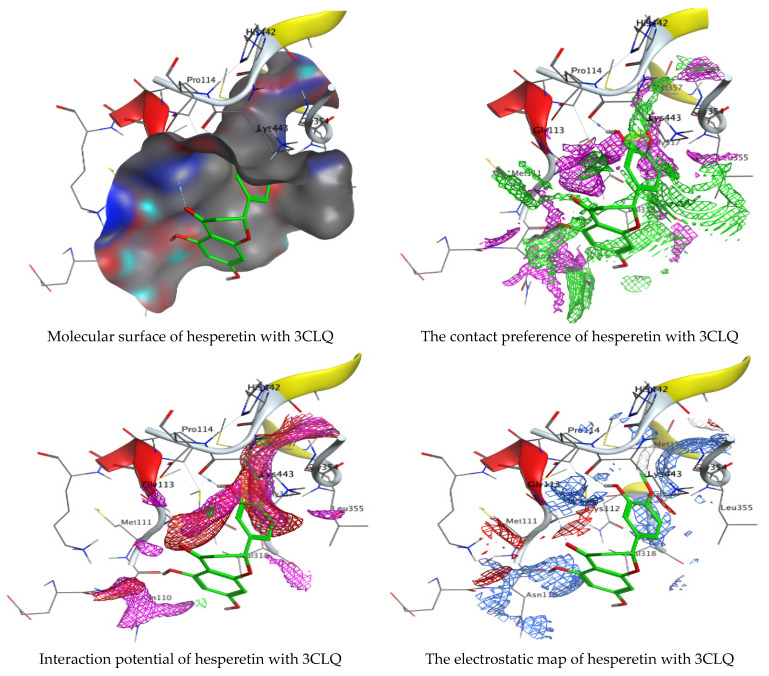
Molecular docking process of hesperetin with 3CLQ.

**Figure 11:**
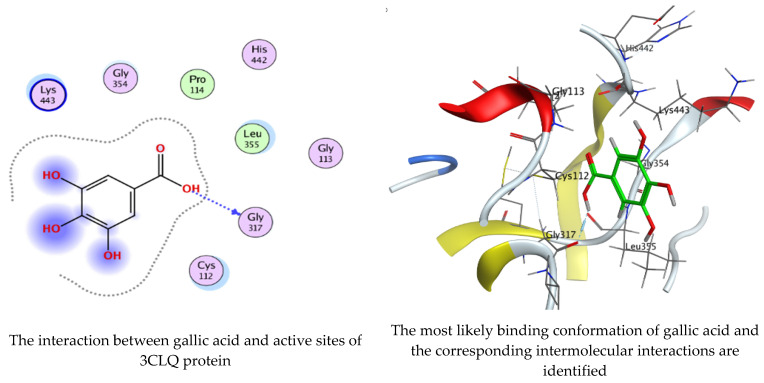

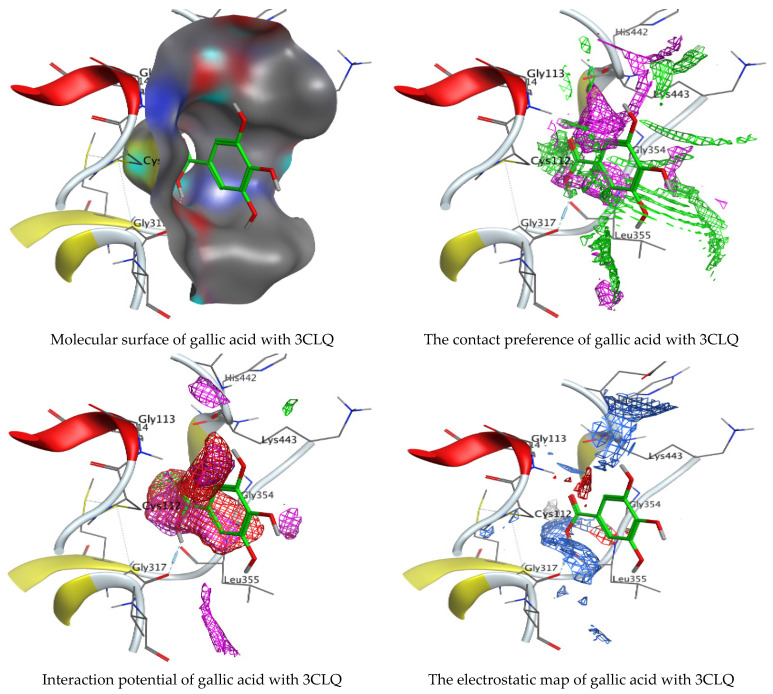
Molecular docking process of gallic acid with 3CLQ.

**Figure 12:**
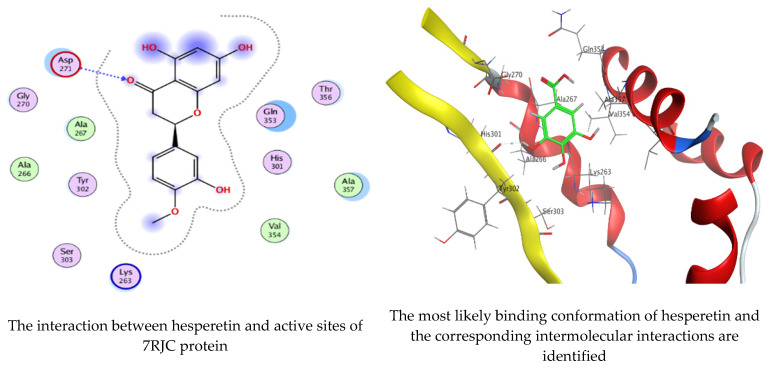

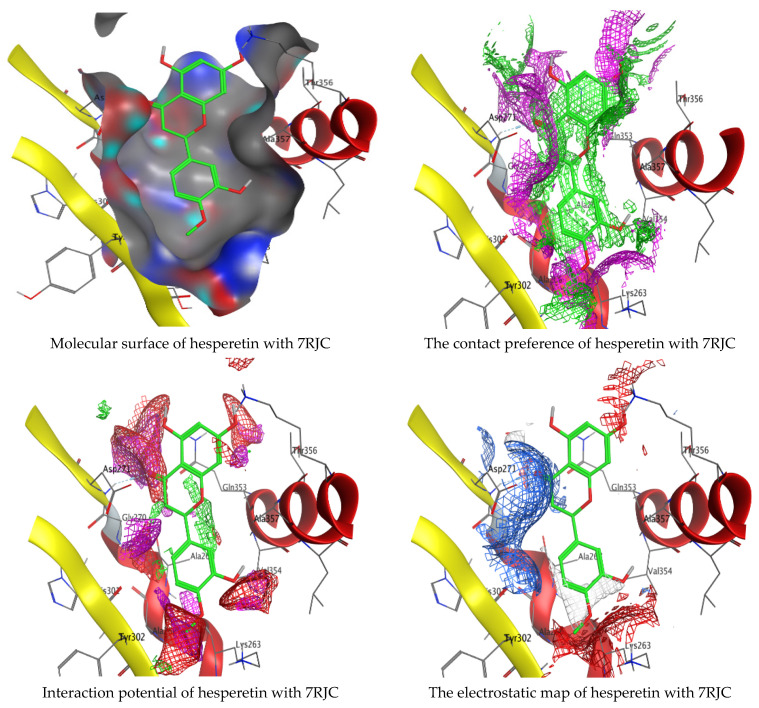
Molecular docking process of hesperetin with 7RJC.

**Figure 13:**
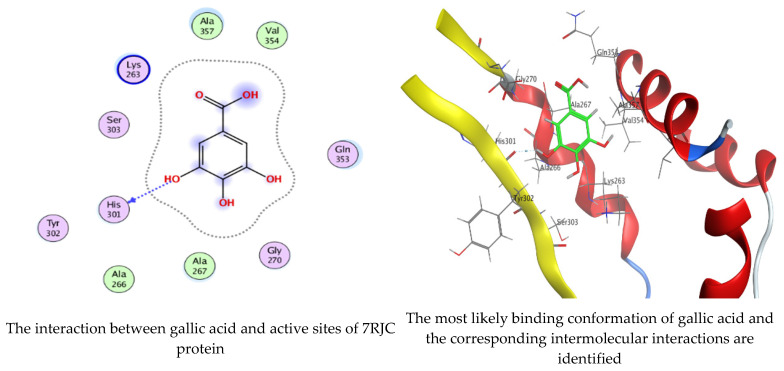

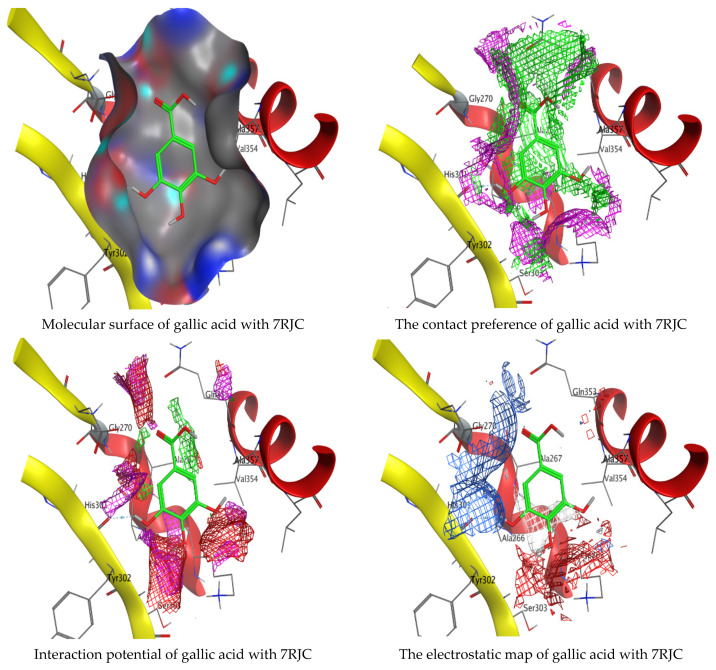
Molecular docking process of gallic acid with 7RJC.

**Figure 14:**
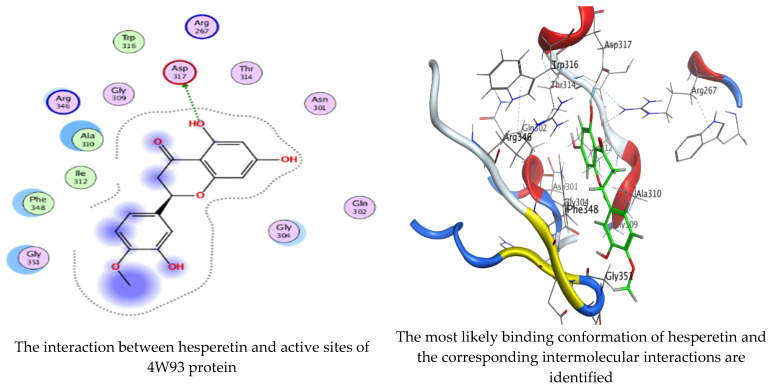

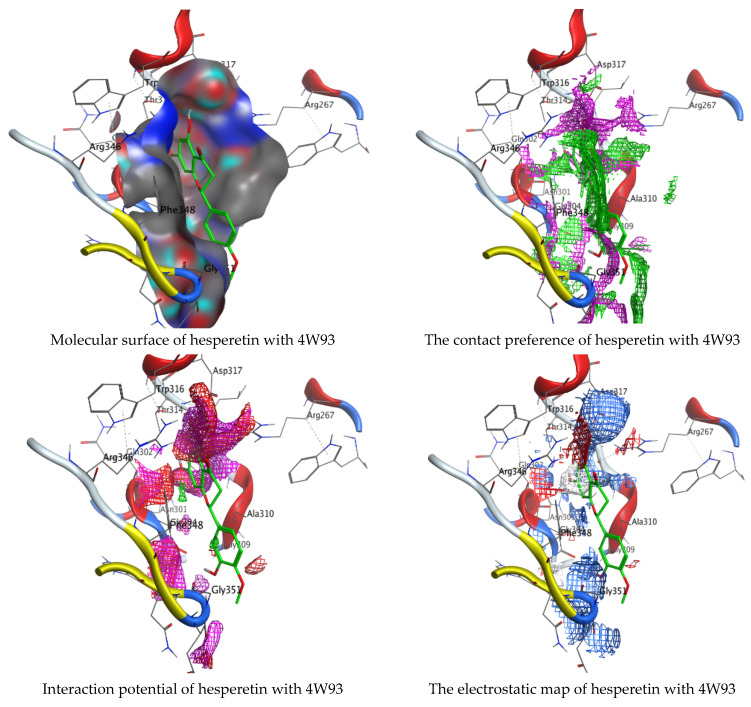
Molecular docking process of hesperetin with 4W93.

**Figure 15:**
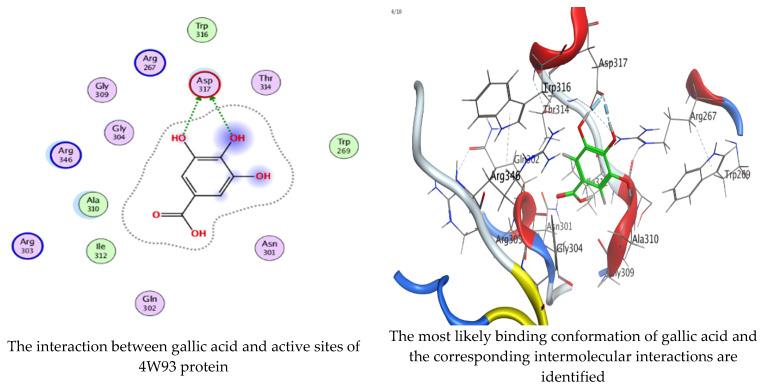

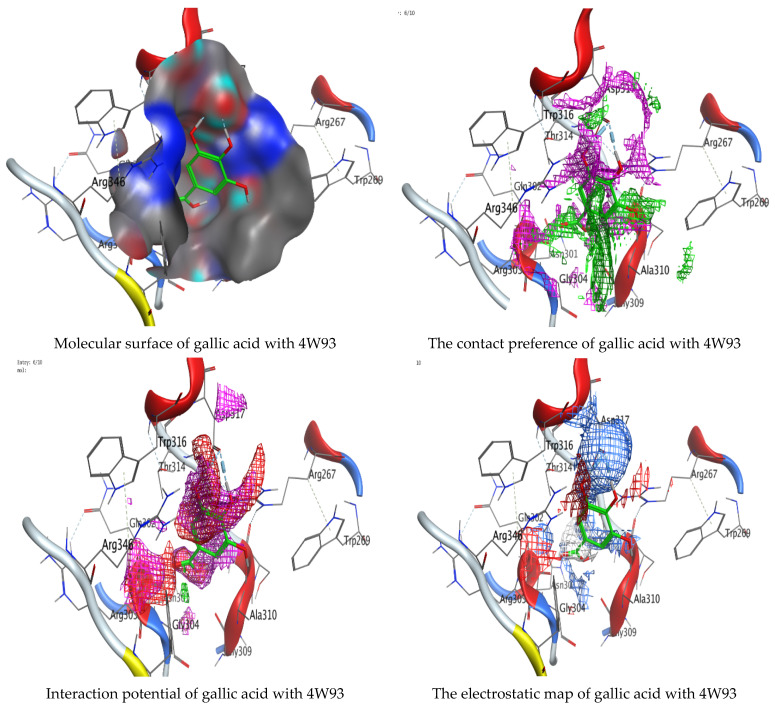
Molecular docking process of gallic acid with 4W93.

**Figure 16:**
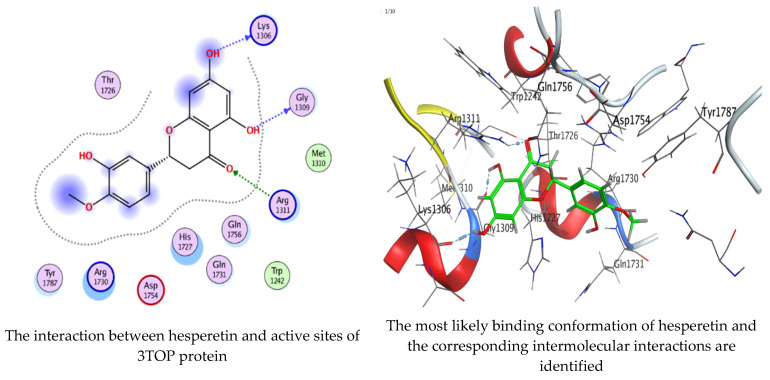

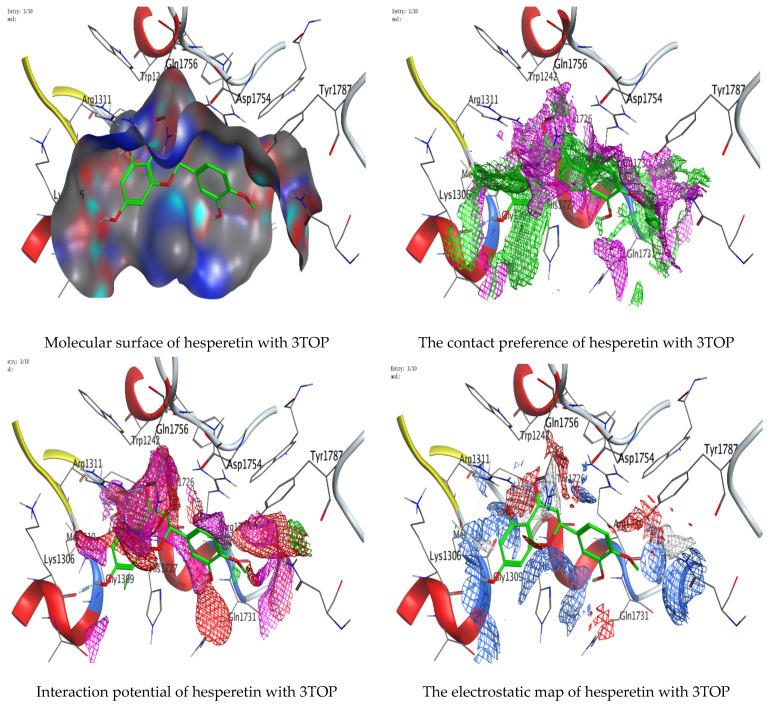
Molecular docking process of hesperetin with 3TOP.

**Figure 17:**
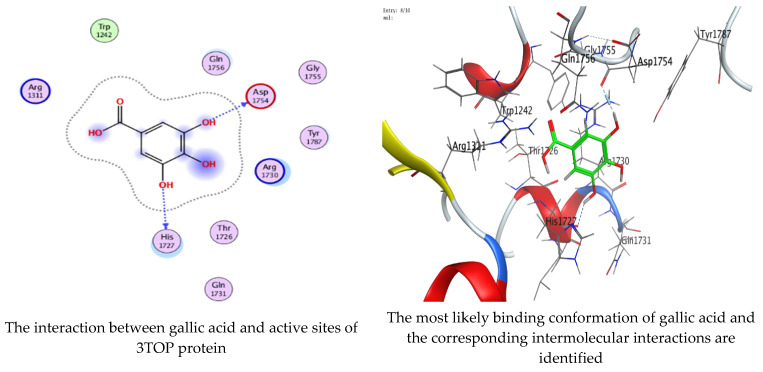

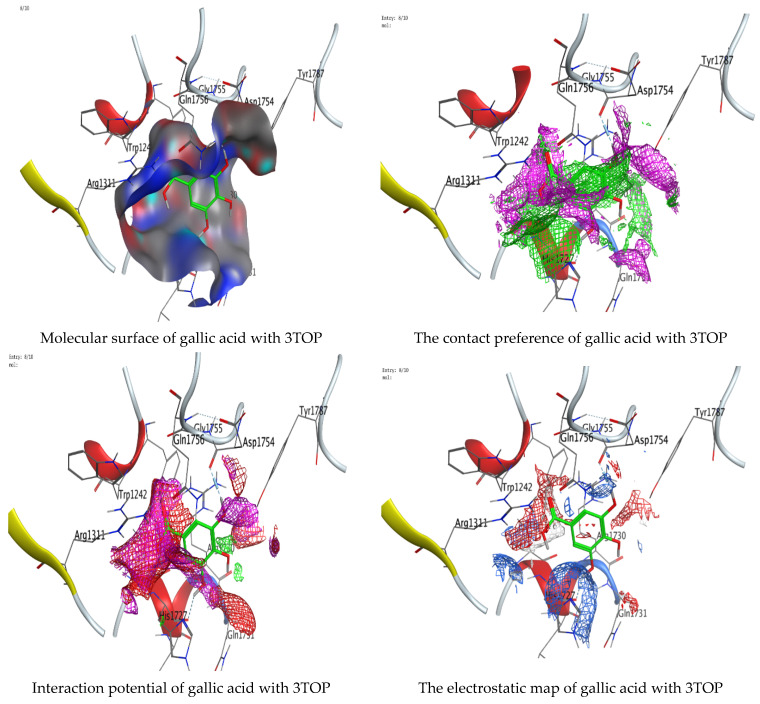
Molecular docking process of gallic acid with 3TOP.

As mentioned in the phenolic and flavonoid analysis of of *C. sempervirens* extract via HPLC, some active constituents of *C. sempervirens* extract, represented by the highest detected flavonoid (hesperetin) and phenolic (gallic acid), were docked with some tested microorganisms protein, including *E. faecalis* (PDB ID: 3CLQ) and *C. albicans* (PDB ID: 7RJC), as well as with α-amylase (PDB ID: 4W93) and α-glucosidase (PDB ID: 3TOP), to support the activity of the extract. Several studies applied molecular docking tools to confirm the activity of natural compounds, discover new ones, or design pharmacological compounds for the treatment of numerous diseases [5,20]. The results showed that both ligands studied have a comparable position and orientation inside the putative binding site of proteins, revealing a vast region limited by a membrane binding domain that serves as an entrance conduit for the substrate to the active site (Figure 10, Figure 11, Figure 12, Figure 13, Figure 14, Figure 15, Figure 16 and Figure 17). Furthermore, the affinity of any small molecule can be thought of as a unique instrument in the realm of drug design. The affinity of organic molecules and the free energy of binding have a relationship. This relationship can help anticipate and interpret the activity of organic chemicals towards given target proteins. The selected compounds had favorable binding free energies values in negative Kcal·mol^−1^ (E_score2), as indicated in Table 3. The proposed binding mode of hesperetin revealed an affinity value of −6.3585, −6.25187, −5.92382, and −5.42187 Kcal·mol^−1^ towards α-glucosidase (PDB ID: 3TOP), *C. albicans* (PDB ID: 7RJC), α-amylase (PDB ID: 4W93), and *E. faecalis* (PDB ID: 3CLQ), respectively. While the proposed binding mode of gallic acid reported an affinity value of −4.69897, −4.61066, −4.47121, and −4.20859 Kcal·mol^−1^ against 4W93, 3TOP, 7RJC, and 3CLQ, respectively. The obtained results of molecular docking interactions reflected a low energy score with the 3TOP, 7RJC, 4W93, and 3CLQ proteins indicating the vital role of hesperetin for repressing bacteria and enzymes action if compared to energy score in the case of gallic acid. In the study of the binding properties of hesperetin the O 26, O 30, and O 25 atoms form hydrogen bond contact with the ASN110, LYS443, and GLY113 amino acid residues of *E. faecalis* 3CLQ, respectively. The O 25 atom bonded with the ASP271 amino acid residue of *C. albicans* 7RJC. The O 26 forms donor hydrogen bond with the ASP317 of α-amylase 4W93. Finally, hesperetin formed three interactions with GLY1309, LYS1306, and ARG1311 of α-glucosidase 3TOP through its O 26, O 28, and O 25 atoms, respectively.

On the other hand, gallic acid inhibits *E. faecalis* 3CLQ through binding directly the O 17 atom to the essential amino acid inside its pocket (GLY317). The O 11 atom forms hydrogen bond with HIS301 amino acid of *C. albicans* 7RJC, where O 9 and O 13 undergo hydrophobic contact with ASP317 amino acid residue of α-amylase 4W93. There is a hydrogen bond observed between (O 11, O 13) atoms and (HIS1727, ASP1754) in α-glucosidase 3TOP respectively. Table 4, Table 5, Table 6 and Table 7 illustrate several interactions with amino acids in the target proteins pocket. They were stabilized at the protein binding site by altering multiple electrostatic bonds, which was reflected in the biological activity profile. The binding energy among the active constituents and the target receptor proteins resulted from molecular docking analysis encouragements a possible antimicrobial and antidiabetic role of *C. sempervirens* extract. Previously, Zengin et al. [11] mentioned that the best docking pose resulted from the docking interaction of apigenin, protocatechuic acid, and chlorogenic acid and catechin as constituents of *C. sempervirens* extract against the α-glucosidase and α-amylase was mainly stabilized through pi–pi stacks and hydrogen bonds. From recent investigations, ellagic and chlorogenic acids were docked with crystal structures of *C. albicans* (4YDE) and *Geotrichum candidum* (4ZZT), where ellagic acid reflected less docking score (−6.19 and −4.51 Kcal/mol) than chlorogenic acid (7.84 and −5.70 Kcal/mol) against *G. candidum* (4ZZT) and *C. albicans* (4YDE), respectively [4]. According to Molecular Docking and Molecular Dynamic Simulation analysis, *C. sempervirens* is an excellent candidate for SARS-CoV-2 virus management [3].

## 3. Material and Methods

### 3.1. Used Chemicals

Mueller Hinton broth, Sabouraud dextrose broth (MHB, Oxoid, Basingstoke, UK), Acetonitrile, dimethylsulfoxide (DMSO), 2,2-diphenyl-1-picrylhydrazyl (DPPH), methanol (HPLC Grade, purity > 99%), and Mueller–Hinton agar were obtained from Sigma-Aldrich (Steinheim, Germany). Conversely, gentamicin, fluconazole, H_2_SO_4_, sodium phosphate (NaH_2_PO_4_), ammonium molybdate, potassium ferricyanide, ascorbic acid (American Chemical Society Grade), potato dextrose agar medium, and 3,5-dinitrosalicylic acid (Analytical Grade) were obtained from Active Fine Chemicals Limited, Dhaka, Bangladesh.

### 3.2. Collection of Plant Material

Aerial parts including leaves and stems of *Cupressus sempervirens* L. were collected from the garden of El-Orman, Giza, Egypt in august 2023. The collected plant parts were washed with running water, and then dried in air, followed by drying in oven at 50 °C for obtaining constant weight, then powdered, and preserved at 25 °C for phytochemical analysis and further biological activities.

### 3.3. Determination of Phenolic and Flavonoid Components by HPLC-UV Assay

*C. sempervirens* extract was subjected to high-performance liquid chromatography (HPLC) for flavonoid and phenolic analysis. HPLC-(Agilent 1100), fortified with two pumps of liquid chromatography, detector of UV/Vis, C18 column (125 mm × 4.60 mm, 5 µm particle size) (Agilent, Santa Clara, CA, USA). The prepared extract (20 µL) was inoculated in HPLC, with using mobile solvents (acetic acid/water, 60:40 *v*/*v*) for the separation of phenolic acids. Conversely, methanol/water (50:50 *v*/*v*) was used as mobile phase for separation of flavonoids, at rate of 1.0 mL min^−1^. The gradient linear was serially automated into the mobile phase with the following arrangement: 82% A at 0 min, 80% A at 0–5 min, 60% 5–8 min, 12% at 8 min,1 5% at 12 min, 16% at 15 min, and 20% at 16 min. The wavelength detector was adjusted at 280 nm, with keeping the column at 40 °C during the entire run process. The appeared peaks of phenolic and flavonoid constituents were recorded for the identification of the separated compounds via the comparison of its retention time besides UV-Vis spectra with injected standards compounds.

### 3.4. Antimicrobial Activity of C. sempervirens Extract

The antimicrobial activity of *C. sempervirens* extract was ascertained using the well diffusion technique. Each microorganism’s inoculum was made 24 h in advance using Potato dextrose broth for fungi (*Candida albicans* and *Mucor circinelloid*) and Mueller Hinton broth for bacteria (*Enterococcus faecalis*, *Staphylococcus aureus*, *Escherichia coli*, and *Salmonella typhi*). Using a densitometer (BIOSAN, Latvian Republic), the pre-cultured inoculum was adjusted to an optical density of 0.5 McFarland standard at 1.5 × 10^8^ Colony-Forming Units per milliliter (CFU/mL). The inhibition zones resulted from the effect of extract which estimated via measuring of its radii from well edge to zone edge. Dimethyl sulfoxide (0.1%) and 100 µL of 5 µg antibiotic (gentamicin)/antifungal (fluconazole) served as a negative and positive control, respectively. For minimum inhibitory concentration (MIC) detection, Mueller Hinton broth/Sabouraud dextrose broth was used for bacterial/fungal inoculum activation and preparation for 24 h/48 h at 37 °C/25 °C, respectively. With the appropriate broth, the microbial culture was diluted to adjust the inoculum level to an optical density (0.5 McFarland standard). Then, 100 μL of microbial inoculum was inoculated in a 96-well microtiter plate. Via serial dilution, different concentrations of the extract were added to the wells. Media with extract only served as a negative control, while microbial inoculum without extract served as a positive control for obtaining maximum microbial growth. At 570 nm, the absorbance of plates was recorded at 0 h of inoculum time and repeated at the same wavelength after 24 h. Then, the value of MIC was calculated using log it analysis. MIC was recorded for fungal growth via a spread of 100 μL of fungal inoculum (adjusted to 0.5 McF of 1.5 × 10^8^ CFU/mL) on a petri dish containing sabouraud dextrose agar medium. Different concentrations of 7.8 up to 500 μg/mL of the extract were achieved through dilution in DMSO solution (0.1%). In total, 10 μL of each concentration was applied in 6 mm of agar well followed by incubation the fungal culture for 5 days at 25 °C. The zone radii of inhibition (zone without microbial growth around the loaded well with the extract or antimicrobial compounds) were recorded [20]. For measurement of minimum bactericidal concentration (MBC), a pure active selected microbe for overnight was diluted broth MH at 1 × 10^6^ CFU/mL. An inventory dilution of the *C. sempervirens* extract is made at 100% instances of the MIC. Furthermore, equal volume (1:1 dilutions) is made in 96 microtiter plates. All concentrations of the *C. sempervirens* extract are injected with equal volumes of the tested microbes. Some wells were used as a positive and negative control for every tested microorganism to reveal acceptable growth over the appropriate incubation period. The possibility of the MBC is detected by the dilution signifying the MIC and at minimum two more concentrated test product dilutions. The lowest dose that displays a pre-defined lessening in CFU/mL is the MBC. The aspects of utilized *C. sempervirens* extract regarding static or cidal were recorded through the detection the index of MBC/MIC; if the ratio of MBC/MIC is no greater than four intervals of the MIC, the *C. sempervirens* extract has a cidal value [37].

### 3.5. Antioxidant Activity of C. sempervirens Extract

#### 3.5.1. DPPH Free Radical Scavenging Test

The capacity of *C. sempervirens* extract to scavenging the free radical was assessed. In total, 3 mL of DPPH solution (0.1 mM) was added to 0.1 mL of the methanolic extract at different concentrations. At wavelength 515 nm, the absorption was measured. Ascorbic acid was served as standard [36]. The IC_50_ value (the amount requisite to inhibit 50% of the DPPH free radical) of *C. sempervirens* extract was computed using a log concentration of inhibition curve. The following Formula (1) was applied to calculate the scavenging capacity of DPPH radical:

(1) $$DPPH\;inhibition\left(\%\right)=\frac{Absorbance\;of\;control\;reaction-Absorbance\;of\;extract\;reaction}{Absorbance\;of\;control\;reaction}\times 100$$

#### 3.5.2. Total Antioxidant Capacity Assay (TAC)

Using the phosphomolybdenum technique, the extract was evaluated spectrophotometrically in accordance with the steps outlined by Prieto et al. [38]. Three mL of the reagent solution (0.6 M H_2_SO_4_, 28 mM of NaH_2_PO_4_, and 4 mM ammonium molybdate) were combined with one mL of each sample extract (0.5 mg/mL). Only 4 mL of reagent solution made up the blank solution. The mixes were incubated for 150 min at 95 °C. Microtiter plate reader (Biotek ELX800; Biotek, Winooski, VT, USA) absorbance at 630 nm was measured when the mixture had reached room temperature. According to Lahmass et al. [39], values were represented as ascorbic acid equivalent (AAE) g/mg of extracts.

#### 3.5.3. Ferric Reducing Antioxidant Power (FRAP) Assay

The potassium ferricyanide and trichloroacetic acid technique [40] was utilized with various modifications and adaption for the microplate method [41] to examine the effect of solvent polarity on the overall reducing power of extracts. Beyond this point, total reducing power (TRP) will be used instead of ferric reducing antioxidant power (FRAP). In total, 40 mL of sample, 50 mL of buffered sodium phosphate dihydrate (Na_2_HPO_4_. 2H_2_O), 50 mL of 1% potassium ferricyanide K_3_[Fe (CN)_6_], and 50 mL of 10% trichloroacetic acid were added to each labeled Eppendorf tube. For 10 min, the mixture was centrifuged at 3000 rpm. Following centrifugation, 33.3 mL of ferric chloride (FeCl_3_, 1%) and 166.66 mL of the supernatant from each sample were put to 96-well plates. Microtiter plate reader (Biotek ELX800; Biotek, Winooski, VT, USA) readings were collected at 630 nm. Ascorbic acid (1 mg/mL) was utilized as a positive control and DMSO as a negative control. The ascorbic acid equivalent (AAE) mg/mg of extract was used to express the results.

### 3.6. Antidiabetic Activity of C. sempervirens Extract

#### 3.6.1. α-Glucosidase Inhibition

The used extract was tested for α-glucosidase inhibitory activity using a slightly modified version of the procedure described by Pistia-Brueggeman and Hollingsworth [42]. In total, 50 μL of the tested extracts at varied quantities (1.97 to 1000 μg/mL) was mixed with 125 μL of 0.1 M phosphate buffer (pH 6.8) and kept at 37 °C for 20 min with 10 μL of the α-glucosidase solution. In total, 20 μL of 1 M *p*-nitrophenyl-*α*-D-glucopyranoside (*p*-NPG) as enzyme substrate was added to initiate the reaction after 20 min, and the mixture was then kept for 30 min. With the addition of 50 μL of Na_2_CO_3_ (0.1 N), the reaction was stopped, followed by measuring the absorbance at 405 nm using a spectrophotometer (Biosystm 310 Plus). α-glucosidase inhibition was calculated via the following Formula (2):

(2) $$\alpha -Glucosidase\;inhibition\left(\%\right)=100-\left(\frac{AE-AES}{AE}\right)\times 100$$

where AE is the absorbance of α-glucosidase without treatment with the extract and AES is absorbance of α-glucosidase treated with the extract.

One unit of the α-glucosidase was defined as the quantity of α-glucosidase necessitated for the creation of one μmol of p-nitrophenol from the p-NPG per minute.

#### 3.6.2. α-Amylase Inhibitory Activity

The used extract was tested for α-amylase inhibitory activity using a slightly modified version of the procedure described by Wickramaratne [43] using 3,5-dinitrosalicylic acid (DNSA) technique. In total, 10% of DMSO were used to dissolve the tested extract, and then it was further dissolved in Na_2_HPO_4_ (0.02 M)/NaH_2_PO_4_ (0.02 M) and 0.006 M of NaCl adjusted at pH 6.9 as a buffer to obtain ranging concentrations from 1.9 up to 1000 μg/mL. The reaction mixture contained of 200 μL of 2 units/mL of α-amylase with 200 μL of the tested extract, followed by incubation at 30 °C for 10 min. Then, 200 μL of suspended starch in water (1%) was added to the reaction mixture and kept for 3 min. DNSA reagent (200 μL) was added to the reaction mixture (require to stop the reaction) with boiling in a water bath at 90 °C for 10 min. After cooling of the reaction mixture (at 25 °C), it was diluted with 5 mL of distilled H_2_O. Followed by measuring the absorbance at 540 nm using spectrophotometer (Biosystm 310 Plus). The 100% activity of α-amylase as a control 100% was formulated by alternating the tested extract by 200 μL of buffer, while the blank reaction was formulated utilizing the tested extract without the α-amylase solution. α-amylase inhibition was calculated via the following Formula (3):

(3) $$\alpha -Amylase\;inhibition\left(\%\right)=100-\left(\frac{AE-AES}{AE}\right)\times 100$$

where AE is absorbance of α-amylase without treatment (control) with the extract and AES is absorbance of α-amylase treated with the extract.

The concentration extract necessitated to inhibit 50% of the activity of α-glucosidase/α-amylase (IC_50_, μg/mL) was calculated employing regression equation achieved via plotting concentration ranged from 1.95–1000 μg/mL and developed % inhibition.

### 3.7. Coagulation Assay of C. sempervirens Extract In Vitro

The anticoagulant activity of *C. sempervirens* extract was examined via coagulant assays activated partial thromboplastin time (APTT) (unite of time with seconds) and prothrombin time (PT) (time in seconds). Nine/one parts of human blood (from corresponding author of the current paper)/sodium citrate (3.2%) (*v*/*v*) were mixed, and then centrifuged for 10 min at 5000 rpm, followed by supernatant collection containing plasma. Different doses (25, 50, and 75 µg/mL) of *C. sempervirens* extract were dissolved in 0.9% aqueous solution of NaCl. The reaction mixture contained plasma and *C. sempervirens* extract, and then kept at 37 °C for 3 min. The reagent of APTT (0.10 mL) (previously pre-kept at 37 °C for 3 min) was added to the reaction mixture and then kept at 37 °C for 5 min. Later, 0.10 mL of 0.025 mol/L CaCl_2_ (pre-kept at 37 °C for 3 min) was added to the reaction mixture, followed by recording clotting time. For PT test, the plasma was mixed with *C. sempervirens* extract, and then kept at 37 °C for 3 min. The reagent of PT (0.20 mL) (pre-kept at 37 °C for 3 min) was added to the reaction mixture, followed by recording clotting time. The cited method was re-performed with heparin as appositive control and with plasma without *C. sempervirens* extracts as control [44].

### 3.8. Experimental Methods of Molecular Docking Interaction

Molecular operating environment (MOE) software on Dell Core i7 processor 1.9 GH, 16 GB memory with Windows 10, 64-bit operating system was used to conduct molecular docking investigations on hesperetin and gallic acid binding properties in *E. faecalis* (PDB ID: 3CLQ), *C. albicans* (PDB ID: 7RJC), α-amylase (PDB ID: 4W93), and α-glucosidase (PDB ID: 3TOP). Each protein used downloaded from the Protein Data Bank (http://www.rcsb.org/pdb, accessed on 13 September 2023). At first, the protein files were prepped using built-in “Quickprep” function. Then, water molecules and bound ligands were manually deleted from the downloaded proteins. The file of the prepared protein active site was loaded; the docking site was specified as dummy atoms, the placement methodology as triangle matcher, and the scoring methodology as London dG. The refinement methodology was adjusted as a rigid receptor and the scoring methodology as GBVI/WSA dG for selecting the best 5 poses from 100 different poses for each tested molecule. The library of compounds was imported and prepped into MOE database file and dock calculations were run automatically. At the end of the docking processes, the obtained poses were carefully studied, and the ones having the best ligand–protein interactions with the most acceptable RMSD values were selected.

### 3.9. Statical Evaluation

The achieved antimicrobial, antioxidant, anticoagulant, and antidiabetic results were obtained in triplicate, so the results were calculated as mean values of ±standard deviation (±SD).

## 4. Conclusions

Several investigations were planned in the present study, including analytical characterization of phenolic and flavonoid constituents in *C. sempervirens* extract with the estimation antimicrobial, antidiabetic, antioxidant, and anticoagulant activities. *C. sempervirens* extract was richened with several phenolic and flavonoid compounds, such as hesperetin (25,579.57 µg/mL), chlorogenic acid (389.09 µg/mL), naringenin (156.53 µg/mL), and quercetin (97.56 µg/mL), which play a vital role in the biosystems. Pathogenic bacteria as well as fungi were inhibited with *C. sempervirens* extract but with different levels of inhibition depending on the type of microbe ranging from 21 to 35 mm inhibition zone. The values of the MIC and MBC of the extract were low for some microorganisms such as *E. faecalis*, *E. coli*, and *C. albicans*, ranging from 7.80 to 15.62 µg/mL. Antioxidant potential via DPPH, FRAP, and TAC was referred to the antioxidant potential of *C. sempervirens* extract. Moreover, antidiabetic activity in vitro via measuring α-amylase (with IC_50_ of 27.01 µg/mL) and α-glucosidase (with IC_50_ of 19.21µg/mL) inhibition; moreover, anticoagulant through PT and APPT assays were linked to *C. sempervirens* extract. The molecular design was performed to investigate the binding mode of the proposed compounds with *E. faecalis* (PDB ID: 3CLQ), *C. albicans* (PDB ID: 7RJC), amylase (PDB ID: 4W93), and glucosidase (PDB ID: 3TOP). The data obtained from the docking studies were fitted with that obtained from the biological screening. All the tested compounds showed significant inhibitory activities against selected proteins. The obtained results indicated that the active compounds could be useful as a template for future design, optimization, adaptation, and investigation in order to generate more potent and selective inhibitors with higher analogs.

## Figures and Tables

**Table 1:** Components of flavonoids and phenolics identified in the *C. sempervirens* extract.

Component	Retention Time	Area	Area (%)	Concentration (µg/mL)
Gallic acid	3.59	254.25	2.19	1107.26
Chlorogenic acid	4.27	57.45	0.49	389.09
Catechin	4.46	13.46	0.12	153.80
Methyl gallate	5.35	137.75	1.19	360.24
Caffeic acid	6.05	15.21	0.13	62.35
Syringic acid	6.38	24.08	0.21	95.94
Pyro catechol	6.58	343.41	2.96	2922.53
Rutin	6.99	129.41	1.11	1313.26
Ellagic acid	7.21	0.00	0.00	0.00
Coumaric acid	8.69	125.63	1.08	233.58
Vanillin	9.09	216.18	1.86	413.13
Ferulic acid	9.71	3.78	0.03	11.53
Naringenin	10.41	32.53	0.28	156.53
Rosmarinic acid	15.97	26.34	0.23	145.71
Daidzein	15.97	69.83	0.60	203.58
Quercetin	17.31	15.44	0.13	97.56
Cinnamic acid	19.33	191.74	1.65	177.51
Kaempferol	20.55	18.72	0.16	61.39
Hesperetin	21.26	9938.11	85.58	25,579.57

**Table 2:** Antimicrobial activity, MIC, MBC, and MIC/MBC index of the *C. sempervirens* extract.

Tested Microorganism	Inhibition Zones (mm)			MIC (µg/mL)	MBC (µg/mL)	MIC/MBC Index
	Extract	* Antibiotic/Antifungal	** Control			
*E. faecalis*	35 ± 0.2	25 ± 0.2	0.00	7.80 ± 0.5	15.62 ± 1.0	2
*S. aureus*	23 ± 0.4	22 ± 0.1	0.00	125 ± 2.5	125 ± 2.25	1
*E. coli*	33 ± 0.2	20 ± 0.1	0.00	15.62 ± 1.0	15.62 ± 0.5	1
*S. typhi*	25 ± 0.1	21 ± 0.3	0.00	31.25 ± 1.2	62.5 ± 1.5	2
*C. albicans*	32 ± 0.2	29 ± 0.2	0.00	15.62 ± 1.33	15.62 ± 1.33	2
*M. circinelloid*	21 ± 0.2	16 ± 0.3	0.00	125 ± 3.0	500 ± 5.0	4

* Gentamicin/fluconazole served as a positive control. ** Dimethyl sulfoxide (0.1%) served as a negative control.

**Table 3:** Docking scores and energies of hesperetin and gallic acid with structure of *E. faecalis* 3CLQ, *C. albicans* 7RJC, α-amylase 4W93, and α-glucosidase 3TOP.

Mol	Protein	S	rmsd_refine	E_conf	E_place	E_score1	E_refine	E_score2
Hesperetin	3CLQ	−5.42187	1.747535	−23.2777	−35.9618	−10.0029	−25.5451	−5.42187
Gallic acid	3CLQ	−4.20859	1.471674	−33.0233	−37.7896	−6.38937	−14.4935	−4.20859
Hesperetin	7RJC	−6.25187	1.854136	−25.7032	−94.8581	−10.9026	−33.9136	−6.25187
Gallic acid	7RJC	−4.47121	1.27963	−32.0095	−66.3415	−10.6428	−23.225	−4.47121
Hesperetin	4W93	−5.92382	1.377247	−24.2932	−79.725	−12.4299	−31.6749	−5.92382
Gallic acid	4W93	−4.69897	1.040136	−27.5389	−74.3128	−11.4052	−22.7225	−4.69897
Hesperetin	3TOP	−6.3585	2.155518	−24.8734	−100.872	−12.1185	−37.3104	−6.3585
Gallic acid	3TOP	−4.61066	1.322542	−32.9586	−66.2276	−10.376	−24.2049	−4.61066

**Table 4:** Interaction of hesperetin and gallic acid with structure of *E. faecalis* 3CLQ.

Mol	Ligand	Receptor	Interaction	Distance	E (kcal/mol)
Hesperetin	O 26	O ASN 110 (A)	H-donor	3.31	−0.5
	O 30	O LYS 443 (A)	H-donor	3.07	−2.0
	O 25	N GLY 113 (A)	H-acceptor	3.39	−0.6
Gallic acid	O 17	O GLY 317 (A)	H-acceptor	2.85	−3.9

**Table 5:** Interaction of hesperetin and gallic acid with structure of *C. albicans* 7RJC.

Mol	Ligand	Receptor	Interaction	Distance	E (kcal/mol)
Hesperetin	O 25	N ASP 271 (A)	H-acceptor	3.11	−1.7
Gallic acid	O 11	O HIS 301 (A)	H-donor	2.87	−1.4

**Table 6:** Interaction of hesperetin and gallic acid with structure of α-amylase 4W93.

Mol	Ligand	Receptor	Interaction	Distance	E (kcal/mol)
Hesperetin	O 26	OD2 ASP 317 (A)	H-donor	3.04	−3.4
Gallic acid	O 9	OD1 ASP 317 A)	H-donor	2.92	−1.9
	O 13	OD1 ASP 317 (A)	H-donor	2.71	−3.4

**Table 7:** Interaction of hesperetin and gallic acid with structure of α-glucosidase 3TOP.

Mol	Ligand	Receptor	Interaction	Distance	E (kcal/mol)
Hesperetin	O 26	O GLY 1309 (A)	H-donor	2.82	−1.1
	O 28	O LYS 1306 (A)	H-donor	2.96	−3.0
	O 25	NH2 ARG 1311 (A)	H-acceptor	3.21	−1.4
Gallic acid	O 11	O HIS 1727 (A)	H-donor	3.08	−0.5
	O 13	O ASP 1754 (A)	H-donor	2.81	−2.7

## Data Availability

Not applicable.
